# FILLING THE CARE GAP FOR OLDER ADULTS LIVING IN A RURAL COMMUNITY: A NURSING CARE MODEL

**DOI:** 10.1093/geroni/igae098.3013

**Published:** 2024-12-31

**Authors:** Walter Dawson, Dana Womack, Lace Velk, Alexis Fifield, Seiko Izumi

**Affiliations:** Oregon Health & Science University, Portland, Oregon, United States; Oregon Health & Science University, Portland, Oregon, United States; Oregon Health & Science University, Portland, Oregon, United States; Oregon Health & Science University, Portland, Oregon, United States; Oregon Health & Science University, Portland, Oregon, United States

## Abstract

Ensuring older adults living with multiple chronic conditions are able to remain in their own homes and communities for as long as possible is a health policy priority. Registered nurses (RNs) play a key role in assisting older adults manage their health while living in the community. Yet, the substantial contributions of nursing care such as care management and care coordination in achieving better health outcomes and reductions in healthcare costs remains largely invisible to healthcare systems and policymakers alike. To demonstrate value of nursing care and build a model of nursing services that is effective and sustainable, we conducted a study describing nursing services needed by the patients living in community settings with complex care needs and evaluated the impact of these services on outcomes. Nurses delivering community-based nursing service (CBNS) to patients living with multiple chronic conditions in rural coastal Oregon were interviewed between July and December, 2022. Outcome data about health service utilizations and expenditure of patients who received CBNS are collected between November 2023-March 2024. Interview data describe care nurses deliver to keep older adults in the community while managing their health by filling care gaps in the rural community. Preliminary outcome data analysis suggests that CBNS is a potentially effective model helping older adults remain in their own homes and communities. These findings may inform policy development regarding reimbursement to make CBNS delivery sustainable as well as increase the options for providing optimal care for older adults living with chronic conditions across multiple geographic settings.

